# Pulmonary function parameters among marble industry workers in Lahore, Pakistan

**DOI:** 10.12688/f1000research.52749.1

**Published:** 2021-09-17

**Authors:** Imran Maqsood Butt, Tajammal Mustafa, Shahnaz Rauf, Anjum Razzaq, Javaria Anwer

**Affiliations:** 1 Institute of Public Health, Lahore, 54000, Pakistan; 2 Family and Community Medicine Department, College of Medicine, Imam Abdulrahman Bin Faisal University, Dammam, 31952, Saudi Arabia; 3 Al Aleem Medical College, Gulab Devi Teaching Hospital, Lahore, 54000, Pakistan; 4 Fatima Jinnah Medical University, Lahore, 54000, Pakistan

**Keywords:** Pulmonary functions, Spirometry, Marble dust, Marble workers

## Abstract

**Background: **Occupational contact with dust particles is a well-known phenomenon, particularly in developing countries of the world. Crystalline silica present in marble dust is the main etiology of a rising prevalence of obstructive lung diseases in marble stone workers, who are in direct contact with marble dust in the surrounding environment during their regular work.  The purpose of this study was to compare the pulmonary function parameters of workers in marble workshops and age matched healthy individuals in the Lahore District of Pakistan.

**Methods: **The study included 164 male individuals, 82 individuals working in marble workshops and 82 healthy individuals from the same community. Data were collected through in-person interviews using a structured questionnaire after obtaining written consent. A Spiro Lab spirometry for pulmonary function tests was used to identify any change in the lung function parameters. FVC% (forced vital capacity), FEV 1 (forced expiratory volume in first second) and FEV1 / FVC ratio were evaluated.

**Results: **Mean age in the exposed group (marble workers) and non-exposed group (healthy individuals) were 29.92 ± 6.19 and 30.58 ± 6.37 years, respectively. The mean years of work experience of the exposed group was 11.92 ± 5.67 years. A statistically insignificant difference was observed between marble exposed workers & healthy individuals from the demographic variables. Lung function parameters in marble workers exhibited a highly significant (P < 0.001) decrease in FVC%, FEV _1 _& FEV _1 _/ FVC ratio when compared to healthy individuals.

Seventy-one percent of marble workers had abnormal pulmonary parameters whereas 34% of workers had restrictive pulmonary impairment. Marble workers who had worked for more than 15 years had a highly significant risk of developing abnormal pulmonary function (P < 0.001).

**Conclusion: **Continuous exposure to marble dust deteriorates the lung function of marble workers.

## Introduction

Toxic elements, fumes, and respirable dust produced in marble workshops can cause a health-related risk to the workers in different units of marble workshops. Marble is a semi-translucent very fine to coarse grained crystalline rock. It mainly contains calcite, serpentine and dolomite and is mostly used in making monuments, headstones, and floors. Finely ground marble is used as a whitening agent in toothpastes, paint, and paper. ^1, 2^ Marble dust mainly contains free silica, which means “free of elements” as it is not combined with other elements or silicon dioxide (SiO _2_). Silica is a common ingredient of the Earth’s crust ^3^ and can be found in alpha quartz, beta quartz, moganite, granite, slate, sandstone, and keatite, and it is toxic to the human respiratory system. Occupational exposure to dust containing crystalline silica occurs in the stone, granite, construction, mining, metal foundry, ceramic production and glass industries. ^4^ Airborne dust (free silica) is produced in marble workshops during quarrying, grinding, mining, cutting, and polishing activities and is the main causative factor for many occupational lung diseases such as pneumoconiosis, chronic obstructive pulmonary disease (COPD), silicosis and asthma. ^5, 6^

Continuous inhalation of respirable silica causes many diseases including silicosis characterized by inflammation and pulmonary fibrosis. Silica dust particles enter the alveoli, increase the production of inflammatory mediators in the peripheral airway, and cause emphysema. The particles are mainly deposited on airway surfaces where air flow changes direction. Silica particles having a size 0.2 to 2 micrometers get deposited on the walls of the airway and particles less than 0.2 micrometers enter the terminal respiratory epithelial surfaces and finally diffuse into alveolar gas. ^7, 8^ Deposited crystalline silica particles cause respiratory mucosal irritation, mucosal hypersecretion in the large airways, mucosal gland hypertrophy of trachea and bronchi, an increase in the number of goblet cells in small airways, and excessive mucus formation, which results in the formation of a mucous plug in the lumen, and fibrosis of small airways. ^7, 8^ Obstruction in the air flow in airways results in decreased FVC (forced vital capacity), FEV _1_ (forced expiratory volume in first second) and FEV _1_/FVC ratio airways. ^9, 10^ The results of recent research showed a significant association between pulmonary problems and inhalation of silica dust in Bangladesh. ^11^ Previously published data from Australia demonstrated that stone workers working in factories that perform cutting and grinding activities produce higher contents of respirable silica, which is associated with a higher rate of severe silicosis. ^12^ A previous cross-sectional study among Indian stone-crush workers reported a noteworthy decrease in FEV _1_, FVC and FEV _1_/FVC parameters. ^13^ Several previous studies conducted in Austria, Nigeria, and Lebanon demonstrated that longer exposure to occupational dust (silica) as well as smoking leads to a gradual decrease in Pulmonary Function Tests (PFTs). ^14- 16^

In low and middle economic countries, silica associated lung illness remains a major health threat. In China more than half a million silicosis cases were documented between 1991 and 1995 ^17, 18^ and over ten thousand silicosis associated deaths were reported in South African miners. ^19^ Despite better dust control safety measures and advancements in developed countries, there is a constant need to control dust due to a recent pneumoconiosis outbreak in Australia and the USA. ^20, 21^

Risk of developing lung diseases among silica exposed workers was higher with prolonged exposure to respirable silica dust. The incidence of silica associated silicosis was 12% among the workers who worked for 30 years or more. ^22^ Sharma *et al*. reported that silica is a key factor for other autoimmune diseases such as **systemic lupus erythematosus** (SLE), rheumatoid arthritis, systemic sclerosis, Caplan syndrome, and Erasmus syndrome, which derives from silica exposure and cytoplasmic antibody related vasculitis. ^23^

Marble workers are at a greater risk because of illiteracy, low socioeconomic status, poor knowledge of personal protective measures (PPM), lack of safety rules and their enforcement, and exploitation by employers. ^24^

In developing countries such as Pakistan, plenty of evolutionary projects such as roads, flyovers, underpasses, housing schemes, hospitals etc. are commissioned by the local government and federal agencies. These projects demand people working in the marble sector. Vast research has been done in the stone cutting sector globally but data on pulmonary function parameters for marble industry workers is scarce. ^25^ Thousands of individuals work in small to medium sized marble workshops in a poorly ventilated hazardous environment. The marble workers working in marble workshops are among the most neglected and work in poorly organized areas. The present study measured the impact of silica dust exposure on pulmonary function tests of these workers. The objective of the current study was to compare pulmonary function parameters among the workers of Lahore based marble workshops and age matched healthy individuals.

## Methods

The study was carried out in the Ichra and Township areas of the Lahore District, which are heavily populated commercial areas having the majority of small to medium sized marble workshops. The study unit consisted of marble workers working in wet cutting and dry cutting (grinding) units of the marble workshops. The study group (exposed) comprised male marble workers, aged 18 to 40 years inclusive exposed to marble dust for more than one year, and working 30 hours or more per week. Adult male workers with a diagnosed respiratory system disease (asthma, tuberculosis, bronchiectasis), those with thoracic deformity, those who underwent chest surgery, those who had history of pneumothorax, hemoptysis, recent abdominal or eye surgery, known respiratory malignancy, unstable cardiovascular status (recent heart attack, aneurysm), and those who were unfit for spirometry were excluded.

Normal healthy male individuals from the same community residing near each study site having similar socio demographic status but free from respiratory problems were selected as the control (non-exposed) group.

The sample size was calculated with the WHO sample size calculator using the formula:

$$n=\frac{2\sigma^{2}{\left(Z_{1-\alpha /2}+Z_{1-\beta }\right)}^{2}}{{\left(\mu_{1}-\mu_{2}\right)}^{2}}$$

Assuming 90% power of study, 5% significance level, Z _1-β_ = 90% power of study, population variance (σ ^2^) = 0.5329, anticipated mean for study group I ( $\mu_{1}$ ) = 2.77 (Isara *et al.*), ^15^ anticipated mean for study group II ( $\mu_{2}$ ) = 3.14 (Isara *et al.*), ^15^ the calculated sample size (n) for one group was 82. The total required sample size for both groups was 164.

A total of 164 subjects were selected, 82 marble workers (study exposed group) by simple random sampling (lottery method) and 82 healthy individuals (study control group) were selected by convenience sampling.

The study was approved by the Ethical Committee of the Institute of Public Health (IPH), Lahore, vide letter number 478, dated July 23, 2018. The data was collected from February to April 2019. The data collection techniques (clinical examination, questionnaire, and pulmonary function parameters) were similar for both study groups (marble dust exposed workers and healthy individuals). The study procedures were explained to every study subject and a written consent was obtained from each individual who agreed to participate in the study. All participating subjects were interviewed in-person using functional proforma developed by the American Thoracic Society (ATS) ^26^ and the pulmonary function tests of each subject were addressed as outlined in the Medical Research Council Questionnaire ^27^ ( https://mrc.ukri.org/documents/pdf/questionnaire-on-respiratory-symptoms-1986/). Some items of ATS proforma, like some symptoms and family history, were not included in the questionnaire as these did not relate to our study objectives. The first section of the functional proforma captured information on gender, area of residence, date of birth, marital & educational status. While the second section of the questionnaire documented information regarding occupational history, nature of work, working hours, and total duration of work in years. The height in centimeters and weight in kilograms were measured for all study participants. ^28, 29^ Spirometry was performed by using a Spirolab spirometer (MIR SRL, Roma, Italy). Spirolab spirometry was used throughout the study period for both study groups for PFTs. Spirometry pre-requisites (any potential contraindication, strenuous exercise an hour before test, consumption of bronchodilators) were followed in accordance with the standard protocols and guidelines by ATS. ^30^ The purpose of spirometry and the procedure were explained to each participant. ^30, 31^ The spirometry values obtained were reported with correction for body temperature at ambient pressure, saturated with water vapor (BTPS). Lung volumes (FVC and FEV _1_) were measured in a standing position from a sequence of a minimum of three readings, having adequate start. After probing the data from whole usable curves, the highest FVC & FEV _1_ were noted even though they didn’t belong to the same curve. The FEV _1_/FVC was also calculated from the same tracing.

Spirometry designates the presence of pulmonary impairment, if any of the following recordings were observed ^32^:

• FEV < 80% - predicted normal
• FEV _1_ < 80% - predicted normal
• FEV _1_/FVC ratio < 0.7

The percentages of the predicted values were calculated by Bellamy *et al.*’s following formula ^32^:

FVC (Reading/Predicted Values) × 100% = %age of predicted value

FEV _1_ Reading/(Predicted Values) × 100% = %age of predicted value

FEV _1_/FVC Reading/Reading %

The airflow obstruction can be defined as follows according to the NICE COPD guideline ^33^:

• Mild airflow obstruction - FEV _1_ more than 80%
• Moderate airflow obstruction - FEV _1_ between 50–80%
• Severe airflow obstruction - FEV _1_ between 30–49%
• Very Severe airflow obstruction - FEV _1_ <30% predicted

Obstructive Type Impairment: is characterized by a complete or partial narrowing of airways at any point, which results in an increased resistance and air flow. ^34^ Narrowing of airways is the main reason of obstructive type impairment and is typically found in asthma and COPD ( http://www.irishthoracicsociety.com). Restrictive Type Impairment: The lung capacity is lower than the predicted value for age, sex & size. In this type of impairment, the capacity of lung volume is lower because the lungs are firm & less compliant. There is a decrease in the level of lung parenchymal mobility or chest wall. ^34^ Pneumoconiosis and pulmonary fibrosis are examples of restrictive types of impairment, which causes scarring (fibrosis) of the lungs. Combined Restrictive/Obstructive: In combined type of impairment, all the values of FVC, FEV _1_, & FEV _1_/FVC are decreased. It may be associated with two conditions i.e. asthma and another lung pathology or some pulmonary conditions ( http://www.irishthoracicsociety.com).

**Figure 1.**
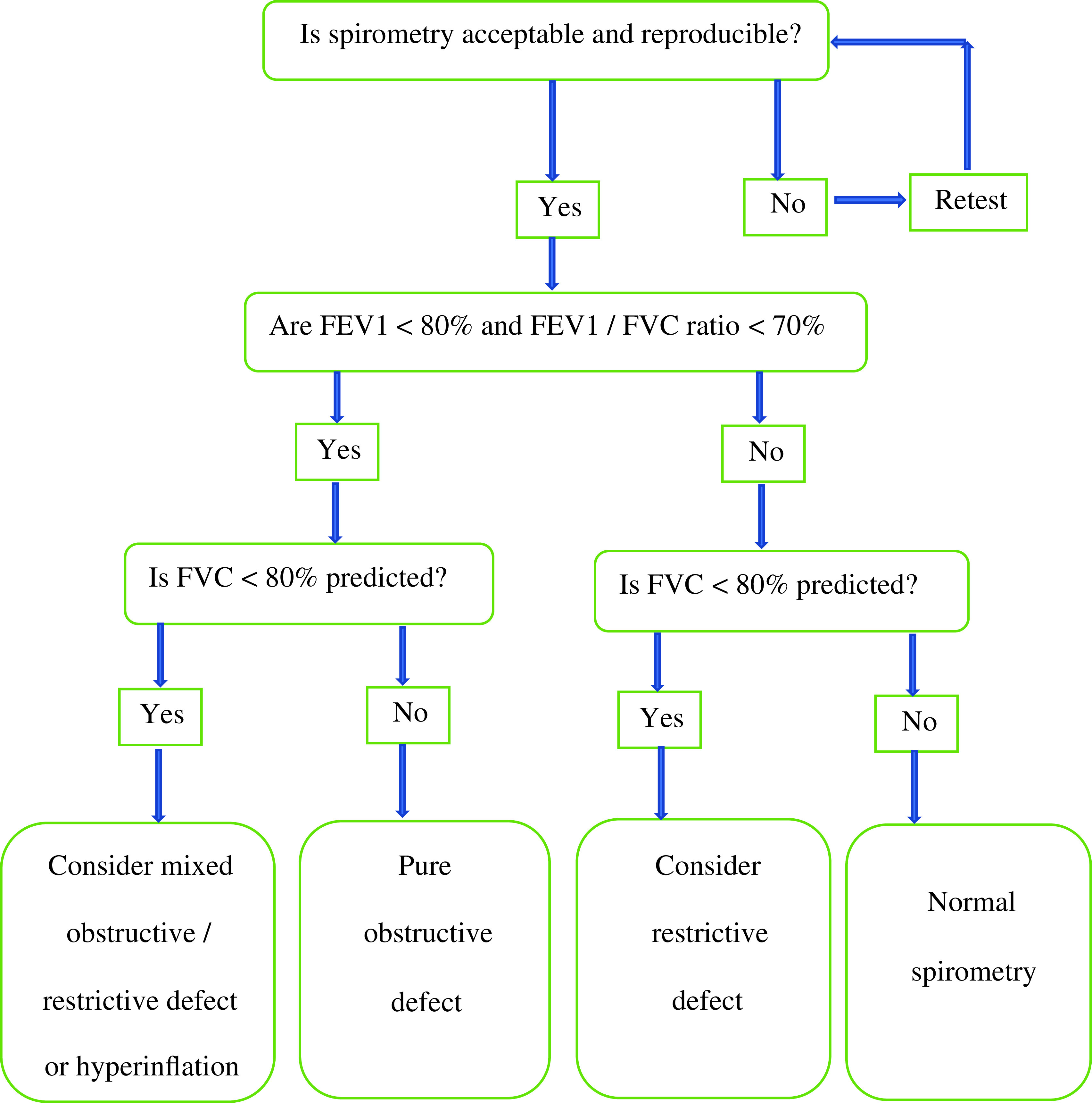
Spirometry diagnostic flow chart – operational definitions of pulmonary impairment.

Figure 1 presents operational definitions of pulmonary impairment as recommended by spirometry flow chart for diagnosis (reference). The underlying study data ( Table 1: Data Marble Feb 19 2021.sav) is available at Figshare. ^35^

**Table 1.** Demographic characteristics of marble workshop workers (Exposed Group) and healthy individuals from the community (Non-Exposed Group).

Study variables	Marble workshop workers (Exposed Group) N (%)	Healthy individuals from the community (Non-Exposed Group) N (%)
**Age (years)** 21–25 26–30 31–35 36–40 Mean ± SD	22 (26.83) 25 (30.49) 14 (17.07) 21 (25.61) 29.92 ± 6.19	18 (21.95) 27 (32.93) 13 (15.85) 24 (29.27) 30.58 ± 6.37
**Marital status** Single Married	43 (52.44) 39 (47.56)	50 (60.98) 32 (39.02)
**Educational status** Middle Matric Intermediate Graduation	63 (76.83) 10 (12.20) 7 (8.54) 2 (22.44)	4 (4.88) 7 (8.54) 28 (34.15) 43 (52.44)
**Smoking status** Smoker Non-smoker	16 (19.50) 66 (80.50)	4 (4.90) 78 (95.10)
**Height (cm)** Mean ± SD	172.84 ± 10.20	170.45 ± 9.56
**Weight (kg)** Mean ± SD	74.39 ± 13.77	74.89 ± 12.56

The data were analyzed in SPSS version 24 and organized in tabulated form according to the frequency distribution table. The continuous variables such as age, duration of dust exposure, pulmonary function values were summarized in mean, median, standard deviation (SD), and categorical variables like nature of work were depicted as frequencies and percentages. The SD was calculated to understand the variation in the study data. The student t-test was employed to compare mean pulmonary function indicators (FVC, FEV _1_, and FEV _1_/FVC ratio) between marble workshop workers and healthy community controls. The ANOVA was applied to compare pulmonary function indicators (FVC, FEV _1_, and FEV _1_/FVC ratio) by years of exposure; Tukey’s method was employed for post-hoc pairwise comparisons. ANCOVA was employed to control and test for confounding due to smoking on pulmonary function indicators. Chi-square test was used to test the difference in the proportion of smokers and pulmonary impairment between marble workshop workers and healthy community controls. The p-value of 0.05 or less was taken as statistically significant for all analyses.

## Results

The current study showed that the average age, height, and weight of the study exposed group (participants exposed to marble dust) were 29.92 ± 6.19 years, 172.84 ± 10.2 cm and 74.39 ± 13.77 kg respectively and for the control group (non-exposed group) were 30.58 ± 6.37 years, 170.45 ± 9.56 cm and 74.89 ± 12.54 kg respectively. There was no statistically significant difference in the sociodemographic characteristics among marble dust exposed workers and the control group (p > 0.05). About 89% of the exposed group had education levels up to matriculation or below, as compared to 13.4% of the non-exposed group (statistically significant with p < 0.001). In the study exposed group, 48% of the subjects were married compared to 39% of participants among the non-exposed group (p = 0.353). Among the exposed group, 19.5% were smokers compared to 4.9% of non-exposed (healthy individuals), and this was statistically significant (p = 0.007) ( Table 1).

About 71% of workers from the exposed group had statistically significant (p < 0.001) abnormal spirometry results whereas none of the healthy individuals (non-exposed) had abnormal spirometry results. The vast majority of lung impairments were of the restrictive type 40.25%; including mild (34.15%) and moderate (6.1%) restriction respectively followed by obstructive type 25.62% of impairment; which includes mild obstruction (10.98%), moderate obstruction (7.32%), moderate-severe obstruction (6.1%), and very severe obstruction (1.22%) respectively, while combined type of impairment was 4.88%.

**Figure 2.**
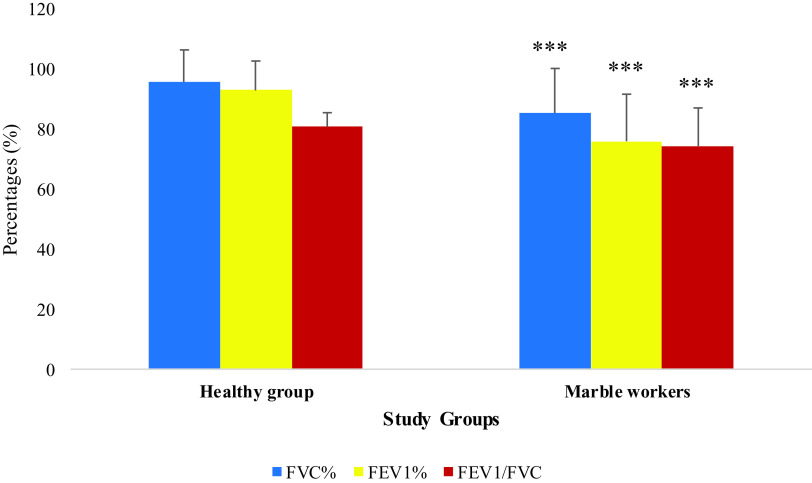
Comparison of pulmonary function parameters (PFTs) by study group (n = 164). Comparison of PFTs (mean ± SD) in study population. *p-value ≤ 0.05, **p-value ≤ 0.01, ***p-value ≤ 0.001 vs healthy group.

The mean values ± SD of FVC%, FEV _1_% and FEV _1_/FVC ratio of non-exposed (healthy individuals) were 95.68 ± 10.66, 92.85 ± 9.78, and 80.73 ± 4.71 respectively and in the marble dust exposed group were 85.36 ± 14.80, 75.93 ± 15.65 and 74.34 ± 12.68, respectively ( Table 2). The FVC%, FEV _1_%, and FEV _1_/FVC ratio for participants exposed to marble dust were significantly lower than healthy individuals (p < 0.001) ( Figure 2).

**Table 2.** Comparison of pulmonary function parameters (PFTs) by study group (n = 164).

PFTs	Marble workshop workers (Exposed Group) n = 82	Healthy individuals (Non-Exposed Group) n = 82	p-value
	Mean ± SD	Mean ± SD	
FVC%	85.37 ± 14.80	95.68 ± 10.66	<0.001 *
FEV _1_%	75.94 ± 15.66	92.85 ± 9.78	<0.001 *
FEV _1_/FVC ratio	74.34 ± 12.68	80.73 ± 4.71	<0.001 *

FVC = Forced vital capacity; FEV = Forced expiratory volume in first second.

* Statistically significant at α ≤ 0.05.

Comparison of pulmonary function parameters (mean ± SD) in the study population showed the average duration of work in marble workshop workers was 11.2 ± 5.677 years, and 51.2% had been working for more than 10 years ( Table 3).

**Table 3.** Distribution of duration of work among marble workshop workers (n = 82).

Duration of work (years)	Marble workshop workers (Exposed Group) (n = 82) Mean ± SD
1–5 6–10 11–15 16–20 Mean ± SD	18 ± 21.95 22 ± 26.83 23 ± 28.05 19 ± 23.17 11.2 ± 5.67

All three pulmonary function parameters (FVC%, FEV _1_% and FEV _1_/FVC ratio) among marble workers by years of exposure showed a statistically significant reduction with increase in duration of work (p < 0.001). The maximum reduction in pulmonary function indices was observed among marble workers who had been working for 16–20 years ( Table 4).

**Table 4.** Results of pulmonary function parameters among marble workers by years of work exposure (n = 82).

Duration of work exposure (years) in marble workshop						
Pulmonary function parameters	1–5 (yrs) (n = 18) Mean ± SD	6–10 (yrs) (n = 22) Mean ± SD	11–15 (yrs) (n = 19) Mean ± SD	6–20 (yrs) (n = 23) Mean ± SD	F	p-value
FVC%	95.90 ± 16.10	84.14 ± 12.21	82.74 ± 14.20	80.48 ± 13.28	7.683	<0.001 *
FEV _1_%	92.36 ± 17.73	73.35 ± 13.38	73.44 ± 7.11	67.62 ± 11.94	14.106	<0.001 *
FEV _1_/FVC ratio	80.81 ± 6.31	73.39 ± 13.60	75.52 ± 13.60	69.20 ± 13.02	6.077	<0.001 *

FVC = Forced vital capacity; FEV = Forced expiratory volume in first second; SD = standard deviation.

* Statistically significant at α ≤ 0.05.

Table 5 shows pairwise comparison of pulmonary function indices among marble workers with years of work in marble industry categories. Compared to participants with 1–5 years’ work exposure, the FVC% and FEV _1_% were significantly decreased among individuals with 6–10 years’ work exposure, individuals with 11–15 years’ work exposure, and individuals with 16–20 years’ work exposure (p < 0.001). Compared to participants with 1–5 years’ work exposure, FEV _1_/FVC ratio was significantly less among workers with 16–20 years’ work exposure. No other significant differences between groups were observed.

**Table 5.** Pairwise comparison of pulmonary function parameters among marble workers by years of exposure.

Pulmonary function parameters	Duration of work exposure (years) in marble industry workers				
	Years	1–5 p-value	6–10 p-value	11–15 p-value	16–20 p-value
FVC%	1–5 6–10 11–15 16–20	- 0.006 * 0.005 * <0.001 *	0.006 * - 0.949 0.733	0.005 * 0.949 - 0.979	<0.001 * 0.733 0.979 -
FEV _1_%	1–5 6–10 11–15 16–20	- <0.001 * <0.001 * <0.001 *	<0.001 * - 0.906 0.18	<0.001 * 0.906 - 0.661	<0.001 * 0.18 0.661 -
FEV _1_/FVC ratio	1–5 6–10 11–15 16–20	- 0.234 0.305 <0.001 *	0.234 - 0.999 0.078	0.305 0.999 - 0.192	<0.001 * 0.078 0.192 -

Post hoc (Tukey’s test).

* Statistically significant (p ≤ 0.05).

## Discussion

PFTs are the main tool to help identify pulmonary abnormalities. PFTs also provide information about airways, pulmonary bed integrity, and parenchymal size. Sharma *et al.* concluded in his study that airborne crystalline silica significantly reduced the FVC and FEV _1_ values in workers who were engaged in marble factories when they were exposed to marble dust as an air pollutant. ^23^ These particles cause respiratory irritation mechanically and release of histamine mediators, which leads to obstruction in the airway.

The current study showed a significant reduction (p < 0.001) in mean values of PFT indices (FVC%, FEV _1_% and FEV _1_/FVC ratio) of marble dust exposed workers as compared to the non-exposed (healthy individuals) group. These results were also in agreement with data reported by Ophir *et al.* in his recent study, who found that ultrafine silica particles are strongly associated with and responsible for the deterioration of pulmonary function parameters among stone factory workers. ^36^ In another study, Ullah *et al.* reported that exposure to high concentration silica particles by stone crushing workers significantly affects their lungs. ^25^ Researchers also observed that carbon containing particles have a contrary effect on the dysfunction of pulmonary functions in addition to silica particles.

Ahmed *et al.* documented direct association of marble dust on lung functions in marble factory workers who had constant exposure to silica dust. ^37^ The spirometry values (FVC%, FEV _1_) were statistically significant (p < 0.01) and had similar differences between the control group and marble workers to our study. Furthermore, many previous studies in India, ^38^ Thailand, ^39^ Iran, ^40^ Egypt, ^41^ Nigeria, ^15^ and Libya ^16^ reported similar findings and mean values of pulmonary function parameters were found to be remarkably deteriorated resulting in pulmonary dysfunction.

The inhaled dust particles get deposited in lungs, cause irritation, and cause an inflammatory reaction leading to fibrosis, faulty oxygen diffusion and abnormal lung functions. ^6^ The change in the elasticity and viscosity of mucous influences its clearance and results in a luminal mucous plug, which is responsible for obstruction to the air flow resulting in decrease in FVC, FEV _1_ and FEV _1_/FVC ratio. ^8^

Reduced values of FEV _1_ and FVC are indications of obstructive and restrictive types of lung changes respectively. The deviations of pulmonary function indicators (FVC, FEV _1_, FEV _1_/FVC ratio) are the major characteristic features of restrictive lung disease. ^31^ In our study, 71% of marble workers had abnormal PFTs while 29% had normal PFT values. Among abnormal PFTs, 40.25% had the highest proportion of pulmonary impairment labelled as restrictive lung pathology, which was followed by 25.62% obstructive type, while 4.88% of cases demonstrated a mixed type of impairment. Our current study findings are quite similar to past studies where researchers reported that 20.1% and 24.3% of lung impairments were of restricted type and 1.5% and 5.8% were of obstructive type. ^42, 43^ Similar results (restriction 23.3% and obstruction 6.2%) were also reported in a previous study among stone crushing industrial workers. ^25^ In contrast to our study, notably less restrictive (2.5%) and obstructive (6.7%) types of pulmonary impairment were reported in a recent study on stone crushing workers. ^11^ The major causes of restrictive type impairment are chest wall limitations, neuromuscular disorder, fibrosis of lung parenchyma, and pleural disorder, whereas obstructive type of pulmonary impairment is commonly seen in chronic bronchitis, asthma and emphysema. ^11^

Age of the study subject could be considered an important causal factor for variation in lung functions and it accounts for a 1.41 fold higher incidence rate using a fixed ratio of variation of lung function parameters. ^44^ Previously published data showed that the anthropometric parameters have a strong association with lung function parameters. ^45, 46^ The present study showed an equal distribution of age group among marble dust exposed workers and non-exposed healthy individuals, both groups were insignificant (p < 0.491). Similar studies on marble stone workers found no statistically significant (p > 0.05) differences in the ages of marble workers and control groups. ^47, 48^

In our study, the results of sociodemographic characteristics showed no statistical difference in age (p < 0.491), weight (p < 0.808) and height (p < 0.1274). Our findings for the mean age, height, and weight are similar to previously reported data by Vyas, ^48^ Shaik *et al.*, ^38^ and Dostbil *et al.* ^49^ for case control studies among marble stone workers.

In the current study, only 20 (12.2%) of the total study participants were smokers, while the proportion of marble workers who smoked were 16 (19.50%) only, compared to four healthy individuals (4.90%). The smoking effect on all PFT parameters was statistically not significant (p > 0.05). Similar findings were reported in a previous study, which showed that smoking did not (p < 0.98) affect PFTs ^15^ although other authors, Rathod *et al*., ^50^ Sheikh *et al*., ^51^ in India, Ullah *et al*., in Pakistan, ^25^ and Jaber *et al*., ^42^ in Cairo reported a significant association (p < 0.05) of smoking on pulmonary function impairment, which contradicts our study. In our study the possible reason of these non-significant effects could be due to shorter smoking duration, the low intensity (only four packs per year) of smoking in the study population, a fewer number of study participants, and less inhalation during smoking.

The mean years of work experience of our participants (marble workers) was 11.20 ± 5.67 years, which was statistically highly significant (p < 0.001). The majority of the marble workers (23; 28.05%) were working for a period of more than 16 years and their PFT values markedly decreased due to the high duration of exposure (p < 0.001). The maximum reduction in pulmonary function indices were noted among the marble workers who had been working for 16–20 years. All three pulmonary functions parameters, FVC%, FEV _1_% and FEV _1_/FVC ratio, showed significant reduction with increase in duration of work (p < 0.001).

Our study results are analogues to a previous study in which the study author reported a statistically highly significant (p < 0.001) reduction in PFT values. ^52^ He claimed that the duration of exposure to silica is most important and documented predictor of decreased pulmonary functions in the workers exposed to silica dust.

Our study results are parallel to an earlier study in which the researchers documented a statistically highly significant (p < 0.05) reduction in pulmonary function parameters with longer duration of work exposure in marble factories. ^50^ The researchers also reported that the deterioration of PFTs is strongly associated with increased duration of exposure to silica. ^50^

Similar types of results were also reported among quarry workers in Nigeria where the mean values of PFTs, the negative correlation between longer work exposure and FEV _1_ (p < 0.05), and lower lung function values were reported. ^52^ In concurrence to our study, researchers (Shaik *et al.* ^38^ Nandini *et al.,* ^53^) also reported a significant reduction in FVC, FEV _1_ and FEV _1_/FVC ratio (p < 0.001) in quarry and granite workers having greater than 10 years of silica dust exposure.

There is a possibility of confounding due to age and smoking. Age matched participants from the community (control group) were selected, hence confounding due to age is unlikely. No differences in the pulmonary function test values between smokers and non-smokers were observed. Also, on ANCOVA analysis, smoking was not statistically significant, therefore, confounding due to smoking is also unlikely.

## Conclusion

Pulmonary function parameters were found to be significantly decreased among marble workers who have marble dust exposure compared to healthy individuals from the same community. The current study disclosed that the continuous occupation of the marble industry for more than 15 years resulted in further reduction of workers’ pulmonary function parameters, which ultimately would have harmful effects on their health status.

## Limitations

Exposure to marble dust is not the only reason for pulmonary function deterioration. There could be other possible factors such as toxic materials in the work environment as well as undetectable concentrations of dust particles other than silica. Dust exposed particle sampling was not used to quantify its composition and concentration, and a comparison to silica dust was not made in both study groups. In addition to this, further respiratory procedures like chest radiographs should be carried out for comparison with previous literature in order to verify findings.

## Data availability

### Underlying data

Figshare: Underlying data for ‘Pulmonary function parameters among marble industry workers in Lahore, Pakistan’, https://figshare.com/s/7a24edab6fc0d64c526c. ^35^

This project contains the following underlying data:

• Table 1: Data Marble Feb 19 2021.sav.

Data are available under the terms of the Creative Commons Zero “No rights reserved” data waiver (CC0 1.0 Public domain dedication).

## Consent statement

Written informed consent was taken from each study participant, the informed consent form was in Urdu, the national language. Under the confidentiality section of the informed consent, it was clearly mentioned that confidentiality will be strictly maintained at all levels, and in reporting the results, no personal information will be shared, and data will be reported in aggregate form. Specific permission was obtained for the publication of participants' data.
