# Supplementary material for: Pulmonary function parameters among marble industry workers in Lahore, Pakistan
Source: F1000Res. 2021 Sep 17;10:938. [Version 1] doi: 10.12688/f1000research.52749.1 (PMC8609395; doi:10.12688/f1000research.52749.1)
Supplement: Peer review history

# Peer review: Reviewer response for version 1

**DOI:** 10.5256/f1000research.56065.r94844

**Published:** 2021-11-22

**Authors:** Ejaz Mahmood Ahmad Qureshi

**Affiliations:** 1 University Institute of Public Health, The University of Lahore, Lahore, Pakistan

**COMMENTS**

 It is one of the best efforts made by the authors to conduct such a study on workers of the marble industry. As reported by the authors, the study setting where this study was carried out is thickly populated, located in a residential area, hence named cottage industry where marble crushing/cutting activities have been going on in houses since long ago. Being located in a thickly populated place, lots of people are directly and indirectly exposed to silica dust. It would have been a remarkable effort if particulate matter (PM), both PM 2.5 and PM 10 were also measured as mentioned in observation 3.

 Secondly, as silica particles (being heavy in weight) are suspended in the atmosphere for a short distance around these plants, it would have been a great effort if a pulmonary function test was performed in people living at least 50 meters from the marble industry as noted in observation 1. Respiratory symptoms in these people should also be recorded.

 In addition to that, one of the limiting factors of this study was its short duration. It would have been better if this study was carried out in a larger time period, 6 months or one year, as the weather conditions can affect the outcome of the result. It is usually seen that in hot weather, workers try to stay and work at the point where some fans are plying with the result that dust spread is more in such cases and chances of getting affected is more than in winter season. Technology being used in the marble industry is also very important. In those places where wet technology is used for cutting marbles, the spread of dust particles is far less than in those places where marble is cut in dry conditions.

 Regarding confounding factors like the presence of pollutants around the marble industry, it is pertinent to mention that lots of pollutants, like NOX, SOX, CO _2, _etc, are emitted from other sources, like vehicles, burning of waste, etc., which is very common practice, especially in an industrial area. These pollutants might have augmented bad effects on the lungs of workers of the marble industry and people residing nearby and so should have been measured. In addition to that, few workers were smokers as the result of this study reported, but also many people might have been addicted to other substances prepared locally like opium, niswar, battery, etc., which can also enhance the respiratory symptoms and decrease lung function test. These workers should have been included in the study.

 Looking at the prevailing situation in the industrial area in general and study setting in particular, it is the need of an hour that Environmental Monitoring, namely Environmental Impact Assessment (EIA) for larger marble cutting units and Environmental Examination (EE) for small industries, cottage units by the Environmental Protection Department (EPD) should be done. Although these measures have been in place but unfortunately not strictly followed.  

         

**COMMENTS ABOUT QUALITY OF PAPER**

- **Are sufficient details of methods and analysis provided to allow replication by others?**

Methods of data collection have been given in detail and were quite comprehensive. A list of inclusiveness and exclusiveness is also complete. One can say that this has increased the understanding of the matter even for a layman. Another good thing is the comprehensiveness of data analysis. So, I will say that sufficient details of methods and analysis have been provided and it has added the knowledge of the reader significantly. It is also a very good source of reference for other studies.

- **The status you would give this article:**

I can say with confidence that the article is scientifically valid in its current form. The experimental design, including controls and methods, is adequate; results are presented accurately and the conclusions are justified and supported by the data. Revisions/modifications suggested are minor in nature, and are considered to be non-essential.

Is the work clearly and accurately presented and does it cite the current literature?

Yes

If applicable, is the statistical analysis and its interpretation appropriate?

Yes

Are all the source data underlying the results available to ensure full reproducibility?

Yes

Is the study design appropriate and is the work technically sound?

Yes

Are the conclusions drawn adequately supported by the results?

Yes

Are sufficient details of methods and analysis provided to allow replication by others?

Yes

Reviewer Expertise:

Public Health, Environmental and Occupational Health

I confirm that I have read this submission and believe that I have an appropriate level of expertise to confirm that it is of an acceptable scientific standard.

# Peer review: Reviewer response for version 1

**DOI:** 10.5256/f1000research.56065.r95979

**Published:** 2021-10-29

**Authors:** Shamim Mohammad

**Affiliations:** 1 International Ford Foundation Fellowship, Alumnus, New York, NY, USA

The study "Pulmonary function parameters among marble industry workers in Lahore, Pakistan" is a methodologically sound case-control study. The researchers have taken all the necessary steps to ensure adherence to the application of basic protocols of research. The study finds the negative impact of the long duration of silica dust exposure on the lungs of the marble industry workers. Findings are in line with similar studies conducted around the world on occupational exposure to dust, particularly stone or marble dust. However, a brief justification about the selection of the research site, the absence of female participants in the study, and the incentive provided to the participants, if any, will strengthen the generalizability of the study further.

Is the work clearly and accurately presented and does it cite the current literature?

Yes

If applicable, is the statistical analysis and its interpretation appropriate?

Yes

Are all the source data underlying the results available to ensure full reproducibility?

Yes

Is the study design appropriate and is the work technically sound?

Yes

Are the conclusions drawn adequately supported by the results?

Yes

Are sufficient details of methods and analysis provided to allow replication by others?

Yes

Reviewer Expertise:

Respiratory diseases, mental health, and health inequities

I confirm that I have read this submission and believe that I have an appropriate level of expertise to confirm that it is of an acceptable scientific standard.
